# A Multicentric European Clinical Study on Custom-Made Porous Hydroxyapatite Cranioplasty in a Pediatric Population

**DOI:** 10.3389/fsurg.2022.848620

**Published:** 2022-03-23

**Authors:** Ismail Zaed, Adrian Safa, Piero Spennato, Carmine Mottolese, Salvatore Chibbaro, Delia Cannizzaro, Roberto Faggin, Paolo Frassanito, Rodolfo Maduri, Mahmoud Messerer, Franco Servadei

**Affiliations:** ^1^Department of Biomedical Sciences, Humanitas University, Milan, Italy; ^2^Department of Neurosurgery, IRCCS Humanitas Research Hospital, Milan, Italy; ^3^Division of Neurosurgery, Santobono-Pausilipon Children's Hospital, Naples, Italy; ^4^Department of Pediatric Neurosurgery, Hôpital Femme Mère Enfant, Université Claude Bernard Lyon 1, Lyon, France; ^5^Department of Neurosurgery, Strasbourg University Hospitals, Strasbourg, France; ^6^Division of Pediatric Neurological Surgery, Department of Pediatrics, University of Padua, Padua, Italy; ^7^Pediatric Neurosurgery, Fondazione Policlinico Universitario A. Gemelli IRCCS, Rome, Italy; ^8^Avaton Surgical Group, Clinique de Genolier, Swiss Medical Network, Genolier, Switzerland; ^9^Department of Neurosurgery, University Hospital of Lausanne, Lausanne, Switzerland

**Keywords:** cranioplasty, cranial reconstruction, porous hydroxyapatite, pediatric, cranioplasty complication

## Abstract

**Background:**

Cranioplasty (CP) is a surgical intervention aiming to re-establish the integrity of skull defects. Autologous bone and different heterologous materials are used for this purpose, with various reported related complications, especially in children.This study aims to evaluate the rate of complication in a multicentric cohort of pediatric patients treated by porous hydroxyapatite (PHA) CP implantation and to assess the reliability of post-marketing clinical data collected by a manufacturing company.

**Methods:**

The authors proactively collected clinical data from 20 institutions in different European countries for patients under the age of 16 treated with a PHA implant. The data were obtained by conducting an on-site interview with physicians in charge of the patients (*Post-Marketing Surveillance, PMS group*). The endpoints were the incidence of adverse events and related implant removal. The clinical data were compared to the company-based register including all patients under the age of 16 who received the same implant from January 1, 2004 to December 31, 2020, and the collecting complications voluntarily reported by surgeons *(Database, DB group)*.

**Results:**

The two groups were similar in terms of demographic characteristics and rate of complications. In the PMS group, a total of 11 (16.9%) complications were reported in the group of 65 patients that were proactively collected. Both fractures and infections were the most common complications with 4 cases each (6.2%). In the case of both infections and fractures, revision surgery was required for only one patient (1.5%). Three (4.5%) cases of displacements were reported, and in one (1.5%) case, a surgical revision was required, for a total of 3 (4.5%) cases requiring surgical revision. The average follow-up was 26.7 months.

**Conclusions:**

Different from a previous study on adult age, pediatric neurosurgeons are more prone to report even to the manufacturing company complications related to skull reconstruction in children. Therefore, these data can be compared with those of other clinical studies. The PHA CP in this series of 65 patients presents a complication rate collected on-site that is similar to other heterologous materials.

## Introduction

Cranial reconstruction (CR), also known as cranioplasty (CP), is a common neurosurgical procedure usually performed to restore the cranial vault after decompressive craniectomy or any other previous surgical procedure that affected the skull bone, such as that for erosive tumors. Autologous bone remains the gold standard option for cranial reconstruction; however, this option is not always feasible because of storage limitations and high infection risk in the case of open skull fractures. Recent studies have also shown that these limitations of autologous bone cranioplasty are more important in pediatric patients with a higher rate of bone reabsorption (1, 2). In this setting, it is important to correctly define the pediatric population (3). Children present clinical characteristics that are different from those of the adult population: the growing and dynamic bone remodeling present in kids suggests different surgical outcomes and leads to limitations in the usage of various materials (4). Also, a stratification of age range could be helpful in clinical management and decision (4, 5).

To overcome the limitations of autologous cranioplasty in children, several heterologous materials have been developed recently such as porous hydroxyapatite (PHA), titanium, polyetheretherkone (PEEK), and polymethylmethacrylate (PMMA) (6).

To date, there is still little evidence concerning the optimal material selection for cranioplasty in the pediatric population (3, 7). Among the most recent materials, PHA cranioplasties have gained popularity because of their particular biochemical characteristics that allow for osteointegration with the skull bone. Despite this interest, there are only a few studies that evaluate the use of such devices in the pediatric population.

Furthermore, several complications may occur after PHA cranioplasty in children, such as hematoma, hydrocephalus, displacement, fracture, and infections (8, 9). Two types of complications are identified: (a) related to the procedure itself, and (b) related to the implant material.

In this study, the authors evaluated cranioplasty complication rate in a multicentric cohort of pediatric patients who underwent PHA cranioplasty, and compared these results with a company post-market clinical analysis to see the reliability of self-reported complications by surgeons in the field of pediatric cranioplasty, as previously performed on the adult population (6). Lack of reported complications to the manufacturing company makes it difficult to compare these reports to any other study published by neurosurgical centers.

## Materials and Methods

The study includes patients below the age of 16 years who underwent PHA cranioplasty (CustomBone Service, Finceramica Faenza Spa, Italy).

To achieve the goal of the study, the complication rate in two groups of pediatric patients was compared: the first group included all PHA custom bone implanted patients from Custom Bone Service Fin-Ceramica, Faenza Database; whereas in the second group, a randomly collected group of patients from European Neurosurgical Centers underwent clinical follow-up.

Details about the surgical methodology were already published elsewhere (6). The current European Medical Device Regulation requires post-market clinical follow-up activities to be carried out by manufacturers to evaluate device performance and safety. For CustomBone Service, the activity of post-market clinical follow-up started in December 2018 (10). For this activity, a specific protocol and a case report form (CRF) were produced for data collection. The protocol is included in the Supplementary Material.

Among all institutions using custom-made PHA, 20 neurosurgical European centers have been randomly contacted for data to analyze their experience with this device in pediatric patients. Each center participating in the study was visited by one of the co-authors (IZ) before the coronavirus disease-2019 (COVID-19) restriction period, and CRFs were filled for each patient implanted in the institution with the aid of neurosurgeons involved in the procedure (Supplementary Material). The centers involved in this study are summarized in Table 1. Concerning the use of the database from the manufacturing company, an agreement was reached to allow the authors to use the company database for scientific purposes. The database is web-based, and it contains general information, under a serialized number for privacy purposes, of all patients implanted with a PHA cranioplasty. The company agreed not to influence the collection of data, and the results of the study were not available to the company before study publication.

**Table 1:** Centers involved in the study.

**Country**	**City**	**Hospital**
Belgium	Ghent	Ghent University Hospital
France	Paris	Hôpital Lariboisière
	Strasburg	Hôpital de Hautepierre
Italy	Alessandria	Ospedale SS. Antonio e Biagio e Cesare Arrigo
	Aosta	Ospedale Regionale della Valle D'Aosta
	Bologna	Bellaria Hospital
		Maggiore Hospital
	Brescia	Spedali Riuniti
	Gemona del Friuli	San Michele Hospital
	Naples	Santobono hospital
	Padua	Ospedale Civile
	Palermo	Policlinico Universitario
	Parma	Maggiore Hospital
	Perugia	Santa Maria della Misericordia
	Rome	Bambino Gesù Children's Hospital
	Rozzano	Humanitas Research Hospital
	Udine	Santa Maria della Misericordia
	Vicenza	San bortolo hospital
Switzerland	Lausanne	Centre Hospitalier Universitaire Vaudois
Ireland	Dublin	Temple street children's hospital

Several variables were taken into consideration for this study, namely, demographic information (gender, age, and surgery date), initial pathology, reason for surgery, localization of the cranial lesion, date of surgery, and country. Concerning postoperative outcome, the authors collected all main types of complications related to PHA cranioplasty implantation (deep and superficial infection, device fracture or mobilization, or related to the procedure, i.e., hydrocephalus, epidural, or subdural hematomas). Finally, an explantation and follow-up data were also collected.

No sensitive information was collected during the research, which was limited to the processing of data regarding adverse events and was according to the biomedical device surveillance norms in force (MEDDEV 2.7/1 rev.4) (10).

To reduce the risk of selection bias, despite randomized collection, representativeness of the collected data from the selected centers was compared with the general series of pediatric patients implanted, which was retrieved with the manufacturer's web portal database, an up-to-date, highly protected, and secure ordering platform and working tool in which all the produced devices are regularly recorded (DB group).

The DB group included all pediatric patients who received PHA cranioplasty in different neurosurgical centers from Europe and the United States from January 2004 to December 2020. In the DB group, data on adverse events are reported voluntarily by the surgeon in charge of the patients (1, 2). The representativeness of the collected series in comparison to the entire clinical series has been tested by comparing demographic information (gender, age, and surgery date), initial pathology, line of treatment, and localization of the cranial lesion. Postoperative complications rates in the two groups were also compared.

### Ethics

In the case of a retrospective observational study, no formal Ethical Committees' approval was required. For the required informed consent, see the ethics section. Despite that, all the parts of the study have been conducted according to the Helsinki declaration.

### Statistical Analysis

Continuous variables were analyzed by Fisher's Exact test. Statistical analyses were performed using the Excel software (Microsoft, Redmond, United States). The *P*-values were considered significant if <0.05. To define the representativeness of the clinical series compared to the whole population implanted with the CustomBone device, a *t*-test was performed. A *t*-value > 1.96 was considered significant.

## Results

The PMS group comprehended clinical data from 65 pediatric patients below the age of 16 (for a total of 76 implanted devices) that were collected from the different hospitals visited by the authors. Among all, 36 (55%) of the patients were male and 29 (45%) were female, with a mean age of 9.1 years (1–16 years ± 4.67). They were stratified into four different groups based on the age ranges: there was 1 (1.5%) child in the range 0–2 years old, which has been implanted off-label, 18 (27.7%) children between 2–7 years old, 25 (38.5%) of them were included in the range 8–12 years old, and, finally, 21 (32.3%) patients were in the last group (12–16 years).

The DB group included data from 725 patients (below the age of 16), who received a CustomBone device from January 1, 2004 to December 31, 2020. The majority of devices (98%) were implanted in Europe, mostly, in France, Italy, and Germany.

The comparison between the two groups is summarized in **Table 3**. Results of the PMS group were further detailed.

### PMS vs. DB Group

As reported in Table 2, there was no statistically significant difference in terms of gender (*|t|* = 1.142), cause of decompression (*|t|* = 1.012), or line of treatment (first or second line of treatment) (*|t|* = 1.457). On these grounds, the series of patients proactively collected has been considered representative of the whole implanted pediatric population (Table 2).

**Table 2:** Summary of statistical differences between the clinical series considered in this study and the control group.

	**Series**	**Control group**	**Difference**
**Gender**			NS, *t* = 1.142
Male	55%	62,90%	
Female	45%	37,10%	
**Line of treatment**			NS, *t* = 1.457
First line	75,30%	39%	
Second line	24,70%	31%	
**Surgical revision rate**	4.50%	6,30%	NS, *t* = 0.7433
**Explantation rate**	4,50%	5,60%	NS, *t* = 0.5106

*NS = difference not significant*.

Different from the study performed on the adult population (6), the revision rate (*|t|* = 0.7433) and explantation rate (*|t|* = 0.5106) between the two groups were not significantly different, making the data obtained by self-reporting neurosurgeons in pediatric cranioplasty reliable. Similarly, the rate of complications is summarized in **Table 4**.

### PMS Group

Regarding the etiology of the defect, the majority of pediatrics included in the PMS group were affected by traumatic brain injury (*n* = 39, 60%), while a minority of them also presented with other different issues. In particular, 7 (10.8%) children presented with tumors invading the skull, 6 (9%) of them had vascular pathology, while 6 (9%) others had a congenital malformation. The remaining was presented with infections (3.1%) or other minor pathologies (7.7%). There was no significant correlation between the etiology of the cranioplasty and postoperative surgical outcome.

Forty-three (66%) patients received PHA implantation as the first-line of treatment, while in 22 children (34%), cranial reconstruction was performed as a second-line procedure after the failure of the previous cranioplasty. The patients who received PHA cranioplasty as a second-line treatment were slightly but significantly more prone (*p* =.043) to have postoperative infections. However, no correlation was found between the line of treatment and revision rate.

The site of defect was most frequently fronto-parieto-temporal (*n* = 25, 38.5%), but a great number presented also in the frontal (*n* = 13, 2%) and bifrontal 10 (15.4%) regions. A minority of them were referred for cranioplasty in the frontoparietal (*n* = 4, 6.2%) and parieto temporal (*n* = 5; 7.7%) regions. Finally, 2 (3.1%) patients had localization in the parietal area, and 2 (3.1%) of them in the crown area (Table 3). No correlation was found between the location of the cranial implant and postoperative risk of infection or fracture. Considering the dimensions of the implants, the mean dimension was 22 ± 13 cm^2^. In the 65 patients considered, there was no correlation between the dimension of the cranioplasty and postoperative infections.

**Table 3:** Demographic and epidemiological data.

	**Number**	**(%)**
**Number of patients**	65	100
**Genders**		
Male	36	55
Female	29	45
**Age**		
Mean age	9,09	
0–2 years	1	1, 5
3–7 years	18	27, 7
8–12 years	25	38, 5
13–16 years	21	32, 3
**Initial pathology**		
Traumatic brain injury	39	60
Vascular injury	6	9, 2
Congenital malformation	6	9, 2
Erosive tumor	7	10, 8
Infection	2	3, 1
Other	5	7, 7
**Line of treatment**		
First line	49	75, 4
Second line	16	24, 6
**Localization**		
Bifrontal	10	15, 4
Crown	2	3, 1
Frontal	13	20
Fronto-parietal	4	6, 2
Fronto-parieto-temporal	25	38, 5
Fronto-temporal	4	6, 2
Parietal	2	3, 1
Parieto-temporal	5	7, 7

Overall, a total of 11 (16.9%) complications were reported in the cohort of 65 pediatric patients. Both fractures and infections were the most common complications reported with 4 cases each (6.2%). In case of both infections and fractures, revision surgery was required only in one case (1.5%). Three cases of prosthesis displacements (4.6%) were reported, with one surgical revision (1.5%) required. As such, only 3 cases (4.5%) required surgical revision (Table 4). The average follow-up was 26.7 months.

**Table 4:** Complications reported in the 65 cases.

**Complications**	**Cases (%)**	**Implant removal**	**% removal**
Infections	4 (6, 2)	1	1, 5
Fractures	4 (6, 2)	1	1, 5
Displacement	3 (4, 6)	1	1, 5
**TOTAL**	**11 (16, 9)**	**3**	**4, 5**

## Discussion

The autologous bone flap is the gold standard material for cranial reconstruction due to its characteristics of durability, accessibility, elasticity, low economic costs, and biocompatibility (11). The most common donor areas for autologous bone are the cranium, ribs, and iliac crest. The advantages of using autologous bone include, among others, minimal dislodgement or disintegration due to a higher rate of revascularization and integration with adjacent bone (7, 12). Unfortunately, autologous bone cranioplasty is burdened by a high complication rate, with a risk of bone reabsorption or infection of the autologous flap as high as 51% under 18 years of age (13). This rate is even higher if a lower age threshold is used to define the pediatric population, the risk decreasing linearly with the age of patients (4).

Whenever an autologous bone is not available, several heterologous materials have been proposed in cranial reconstruction, although the level of evidence reached by literature data on the use of these implants remains extremely low, especially in children (7). Thus, a careful evaluation should be made by physicians to choose the appropriate device based on the characteristics of patients.

Among the different options present on the market, one of the most recent is porous hydroxyapatite cranioplasty, which aims to improve biomimetic assimilation using, as the main constituent, a human mineral bone component (12).

Despite significant evolution of the materials and technology used for cranial repair, in the pediatric population, specific additional limitations may occur because of the active stage of growth and development of the cranial vault (14). In a recent systematic review, the overall rate of pediatric CP complications with different synthetic materials was reported to be about 14.2% (7). Titanium graft is reported to have an overall lower complication rate (6.7%) than polymethylmethacrylate (PMMA) and PEEK. The PEEK presents the highest infection rate (16.1%), while PMMA presents a 10.9% total rate of surgical site occurrence: a surgical-site infection rate of 10.9 and 16.4% of graft failure (15). However, the results of different studies are difficult to be compared because of a lack of homogeneity, in particular, concerning the age of patients included in the study (16). Indeed, the demography of case series in the literature presents a large spectrum of cut-off between the pediatric and adult populations, ranging from 13 to 19 years old (4, 5). Recent investigations have shown that cranial development reaches 80% growth at 2 years old, while at 5 years old it is complete for the 90%, allowing for correct brain development (3). Consequently, a critical analysis of the outcome and complications of CP requires proper age stratification of patients. However, the study by Klieverik et al. showed that data in the literature do not show detailed and specific comparable outcomes in children (7). Therefore, long-term follow-up studies on heterologous cranioplasty in the pediatric population would be necessary, with subgroup analysis for specific complications and age stratification to critically compare the results of CP in the pediatric population.

On these grounds, custom-made porous hydroxyapatite showed to be an option for pediatric CP, and literature data allow for stratification of complications by age for PHA implants, with a rate of explantation of 20.8% under 7 years of age, and 6.58% in the group 7–13 years of age (1, 17, 18).

**Figure 1:**
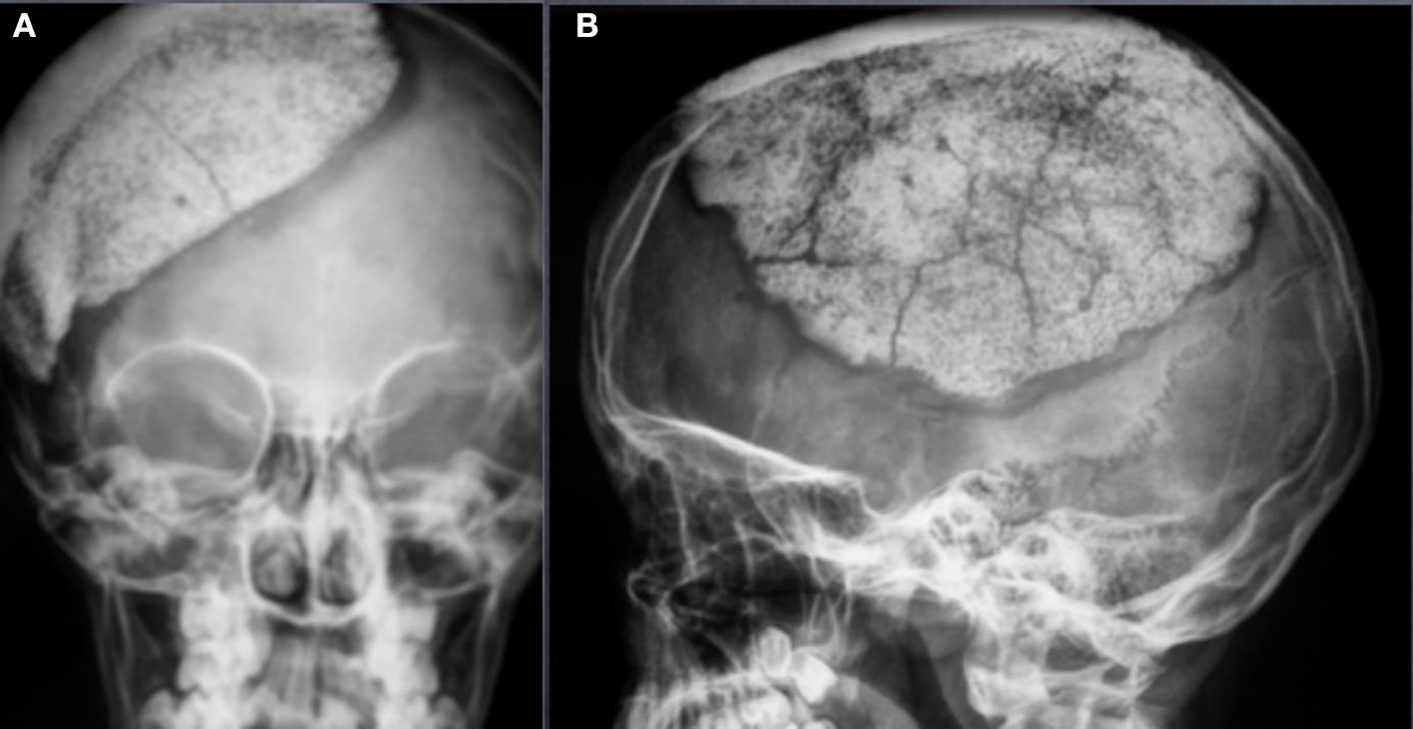
Occipitofrontal (OF) **(A)** and Anteroposterior (AP) **(B)** view of an asymptomatic fracture of a 12 years old patient operated for a decompressive craniectomy after a traumatic brain injury. From the clinical point of view, the patient has no clinical signs of fracture. No surgical revision was needed.

In this study, the main complications reported were fractures (6.2%) (Figure 1), together with infections (6.2%) and displacement (4.6%). In total, 3 (4.5%) surgical revisions were performed.

The rate of fractures in our study is lower than that in other published clinical series (16, 19). This difference in complications could be explained by different factors, among which is a change in the HA cranioplasty manufacturing process. In the beginning, the median porosity of the prosthesis was up to 70% (20), whereas later, it did not exceed 50% (2). Changes were because the presence of porosity allows for better integration with the cranial vault, but it also makes cranial implants more fragile. Therefore, neurosurgeons need to be aware that until a possible bone integration occurs, HA cranioplasty is more fragile than other heterologous materials. Another complication that is worthy of further analysis is a higher rate of displacement compared to the one seen in the clinical series studying other materials (4.6%). In general, heterologous cranial implants are fixed through screws and plates, but this is not possible in the case of PHA cranioplasty because of the risk of prosthesis fracture using screws. Normally, these prostheses are secured with the use of silk sutures through prefabricated holes in implants and this can cause higher mobility of the implants.

Also, causes of the initial pathology resemble the common ones in the literature (21, 22): trauma (60%), tumors (10.8%), vascular (9.2%), malformations (9.2%), infections (3.1%), and others (7.7%). The overall complication rate (16.9%) is similar to what was reported by Klieverik (7).

Furthermore, the present rates of complications and explantation (16.9 and 4.5%, respectively) are comparable to the rates previously reported in children 7–13 years old (14.5 and 6.58%, respectively) (1).

Moreover, the rate of explantation (4.5%) is similar to the rate recently reported in a large adult series of PHA CP (4.25%) (6), although the present study also included patients under 7 years of age at higher risk of complications (23).

The 65 pediatric cases reported here were randomly collected from 20 clinical sites. These cases were treated in most recent years but show similar clinical features of the total pediatric population collected by the manufacturer's database, as confirmed by the comparison between the two groups. The two cohorts of patients were indeed comparable in terms of age, gender, cause of decompression, line of treatment (first- or second-line of treatment), revision rate, and explantation rate (24–26).

Another interesting finding of our study is that different from the adult population (6), the rates of complications in the two groups almost correspond. In our adult series, the complication and explantation rates were almost double in the on-site collected data compared to the company database. This would reasonably fit with higher attention in surveillance and reporting of complications in children by neurosurgeons.

### Study Limitations

Despite the authors' best efforts, this study presents some limitations. One of them is the lack of control comparison of the outcomes among the several synthetic materials used in the cranial reconstruction. Furthermore, the data regarding the timing of cranioplasty, patients' clinical status at surgery, and the presence of systemic infections were not available.

## Conclusions

Our results suggest that pediatric neurosurgeons are more prone to report complications to the manufacturing company; therefore, these data can be directly compared with clinical studies. The complication rate of HA cranioplasty in children age in our series is similar to other heterologous materials. Further clinical comparative studies on heterologous materials in the pediatric age are needed.

## Data Availability Statement

The raw data supporting the conclusions of this article will be made available by the authors, without undue reservation.

## Ethics Statement

Ethical review and approval was not required for the study on human participants in accordance with the local legislation and institutional requirements. Written informed consent to participate in this study was provided by the participants' legal guardian/next of kin. The requesting doctor declares and certifies that the patient, or another individual legitimately authorized to represent the patient, has been given specific information concerning the gathering and handling of personal and sensitive data indicated in this request and the accompanying medical documentation needed for designing, producing, supplying, and post-supply surveillance of the custom-made device in porous hydroxyapatite. Moreover, the requesting doctor declares and certifies that the patient, or other individuals legitimately authorized to represent the patient, has given express consent for the handling of the personal data indicated and for the aforementioned purposes, even by the company involved in the designing, producing, supplying and post-supply surveillance of the device, as well as companies and/or organizations collaborating in the procedure for designing and producing the device proper. Lastly, the requesting doctors declare and certifies that the patient, or other individuals legitimately authorized to represent the patient, has given express consent for the use of the clinical data gathered for scientific purpose.

## Author Contributions

FS and IZ: conception. IZ and AS: data collection. All authors: manuscript drafting. All authors contributed to the article and approved the submitted version.

## Conflict of Interest

FS has received limited grants and consultancy fees from Fin-ceramica spa (producing HA cranioplasty) and Integra-Codman (commercializing HA cranioplasties) and from Johnson and Johnson (commercializing PEEK cranioplasty). PF received consultancy fees from Fin-ceramica spa (producing HA cranioplasty) and Integra-Codman (commercializing HA cranioplasties). Data from this clinical study were elaborated by the authors of this article without any commercial influence. All the remaining authors certified that they have no affiliations with or involvement in any organization or entity with any financial interests (such as honoraria, educational grants, participation in speakers' bureaus, membership, employment, consultancies, stock ownership, or other equity interest, and expert testimony or patent-licensing arrangements), or nonfinancial interests (such as personal or professional relationships, affiliations, knowledge, or beliefs) in the subject matter or materials discussed in this manuscript.

## Publisher's Note

All claims expressed in this article are solely those of the authors and do not necessarily represent those of their affiliated organizations, or those of the publisher, the editors and the reviewers. Any product that may be evaluated in this article, or claim that may be made by its manufacturer, is not guaranteed or endorsed by the publisher.
