# Supplementary material for: A Multicentric European Clinical Study on Custom-Made Porous Hydroxyapatite Cranioplasty in a Pediatric Population
Source: Front Surg. 2022 Mar 23;9:848620. doi: 10.3389/fsurg.2022.848620 (PMC8983879; doi:10.3389/fsurg.2022.848620)
Supplement: Supplementary file 1 [file Data_Sheet_1.PDF]

Patient: (three letters Name, three letter surname) _ _ _ / _ _ _	CustomBoneService Code: ______________________________	Principal prosthesis ☐<br>Back-up prosthesis ☐

Doctor (name/surname) ______________________________

E-mail: ______________________________

Hospital/Clinic: ______________________________

Address Hospital ______________________________

Country: ______________________________

**Data Protection Information**	
The Doctor declares and certifies that the patient or other individual legitimately authorized to represent the patient, has been given the specific consent concerning the gathering and handling of personal and sensitive data indicated in this request and the accompanying medical documentation needed for designing, producing, supplying and post-supply surveillance of the custom-made device in porous hydroxyapatite.	☐
The Doctor declares to be compliant with the provisions of the current legislation regarding the protection of patient's personal data	☐
Doctor signature: ______________________________	Date: ______________________________

PATIENT DEMOGRAPHIC DATA	
Gender	|__| Male |__| Female
Age	|__ __|
Aetiology	☐ Trauma<br>☐ Vascular<br>☐ Tumour<br>☐ Malformation<br>☐ Other
Reason for Device Implantation	☐ Decompression craniectomy<br>☐ Comminuted fracture<br>☐ Tumor resection<br>☐ Autologous bone reabsorption/infection<br>☐ Other material rejection<br>☐ Malformation<br>☐ Other
Localization of Skull Defect	☐ Fronto-parieto-temporal<br>☐ Frontal<br>☐ Fronto-temporal<br>☐ Fronto-parietal<br>☐ Parietal<br>☐ Cranial vault<br>☐ Bifrontal<br>☐ Temporal<br>☐ Occipital<br>☐ Parieto-temporal<br>☐ Other
Side	☐ Left<br>☐ Right<br>☐ Other
Craniectomy Surgery Date	__ __ /__ __ /__ __ __ __ (dd/mm/yyyy)
Cranioplasty Reconstruction Date	__ __ /__ __ /__ __ __ __ (dd/mm/yyyy)
Cranioplasty was performed by Dr.	____________________________
Any intra/post-op AE occurred?	|__| No<br>|__| Yes *(if yes please fill in: Attachment 3: Intra-Op AEs; Attachment 4: Post-Op AEs)*

FOLLOW-UP VISITS	
**FU 30 days**	|__| Yes |__| No
**FU 6 months (1-9 m)**	|__| Yes |__| No
**FU 12 months (9-15 m)**	|__| Yes |__| No
**FU 24 months (15-24 m)**	|__| Yes |__| No
**FU 3 years (+/- 3 m)**	|__| Yes |__| No
**FU 4 years (+/- 3 m)**	|__| Yes |__| No
**FU > 4 years (+/- 3 m)**	|__| Yes |__| No

Patient: (three letters Name, three letter surname) _ _ _ / _ _ _	CustomBone Service Code: ______________________________	Principal prosthesis ☐ Back-up prosthesis ☐

INTRA-OP ADVERSE EVENT		
**Description**		
☐ Implant Device Fracture		
☐ Device does not fit the cranial defect (i.e. because of wrong dimensions/shape of the device)	Specify: __________________________________	
☐ Project error (i.e. mirroring)	Specify: __________________________________	
☐ Surgical error	Specify: __________________________________	
☐ Other surgery-related complications	Specify: __________________________________	
☐ Shipping error	Specify: __________________________________	
☐ Other AE	Specify: ____________________________________	
**Additional Notes**		
Adverse Event Treatment	**Treatment**	
	Yes (please specify how)	☐ Substitution with back-up<br>☐ Surgery resolved with addition of a bone graft<br>☐ Other material (*specify*________________)<br>☐ Other solution (*specify*________________)
	No	☐
	Not yet (please specify why)	☐ Monitoring (i.e. *the device was left in place*)<br>☐ Waiting for a new CBS device<br>☐ No Back Up available<br>☐ The patient's health got worsened (i.e. *swelling*)
AE-Outcome	☐ Resolved<br>☐ Unknown<br>☐ Failure	
Incident	☐ Yes ☐ No	
Relationship with the Device	☐ Related<br>☐ Possibly related<br>☐ Not Related<br>☐ N/A	

Relationship with the Surgery	☐ Related<br>☐ Possibly related<br>☐ Not Related<br>☐ N/A
Is the clinical case part of a Clinical Study?	☐ Yes ☐ No<br>If yes, please provide the following information: |__| Sponsor-Initiated Clinical Study |__| Investigator-Initiated Clinical Study
Has the clinical case been presented to congresses/poster sessions/etc..	☐ Yes ☐ No<br>If yes, please provide information:<br>Congress year ___________________________<br>Congress Event Name _____________________

Further details and comments:

____

____

Date of completion: |__|__| |__|__| |__|__|__|__| Signature: ______________________________

dd mm yyyy

Patient: (three letters Name, three letter surname) _ _ _ / _ _ _	CustomBoneService Code: ______________________________	Principal prosthesis ☐<br>Back-up prosthesis ☐

POST-OP ADVERSE EVENTS					
Did any AE occur?	|__| Yes |__| No				
Adverse Event onset	__ __ /__ __ /__ __ __ __ (dd/mm/yyyy)				
**AE Description**					
☐ Fracture	☐ Spontaneous	☐ After trauma			
	☐ Simple fracture	☐ Comminute			
	☐ Displaced fracture	☐ Other, please specify:________________			
	☐ ≤ 6 months<br>☐ >6 months/ < 1 year<br>☐ ≥ 1 year				
☐ Infection	**Surgical Site Infection**<br>☐ Superficial (skin)<br>☐ Deep: ☐ Meningeal ☐ Medical device ☐ Other<br>☐ Local: ☐ Loco-regional ☐ Systemic				
	**Microbiological exam**				
	☐ bacterial	☐fungus	☐other	☐Unknown	☐ not done
	☐ ≤ 6 months<br>☐ >6 months/ < 1 year<br>☐ ≥ 1 year				
☐ Mobilization	☐ Device Lowering ☐ Device raising ☐ Other: ____________				
☐ Tumour recurrence					
☐ Subdural Hematoma					
☐ Epidural Hematoma					
☐ Wound retraction					
☐ Slow Wound Healing					
☐ Epileptic Seizure					
☐ Other AE	Specify: ____				

**Seriousness**			
☐ SAE - Serious Adverse Event<br>*(any untoward medical occurrence that results in death/is life-threatening/requires inpatient hospitalization or causes prolongation of existing hospitalization/results in persistent or significant disability/incapacity/is a congenital anomaly/birth defect/requires intervention to prevent permanent impairment or damage*			
☐ AE - Adverse Event			
**Severity**			
Grade 1	Asymptomatic or mild symptoms; clinical or diagnostic observations only; no intervention indicated		☐
Grade 2	Moderate; minimal, local or non-invasive intervention		☐
Grade 3	Severe: Symptom(s) causing severe discomfort and significant impact of the patient's usual activity and requires treatment; hospitalization or prolongation of hospitalization indicated; disabling		☐
Grade 4	Life threatening: urgent intervention indicated.		☐
Grade 5	Death related to an AE		☐
**Additional Notes**			
**Was the Device explanted?**			
☐ Yes	☐ No		
AE-Treatment	Yes<br>(please specify how)	☐ Reoperation with back-up CustomBone prosthesis<br>☐ Reoperation with new CustomBone prosthesis<br>☐ Reoperation with the same CustomBone prosthesis (repositioning)<br>☐ Treated in situ with antibiotics (in case of infection)<br>☐ Reoperation with other materials/cranioplasty devices<br>☐ Hospitalized or prolonged hospitalization<br>☐ No other reoperation needed<br>☐ Monitoring<br>☐ Unchanged	
	No	☐	
	Not yet (please specify why)	☐ Patient monitoring ("wait and see")<br>☐ no other reoperation needed	
AE Outcome	☐ Resolved<br>☐ Unknown<br>☐ Failure		

Event classified as Incident	☐ Yes ☐ No
Relationship vs Device	☐ Related<br>☐ Possibly related<br>☐ Not Related<br>☐ Not clear
Relationship with the procedure / surgery	☐ Related<br>☐ Possibly related<br>☐ Not Related<br>☐ Not clear
Is the clinical case part of a Clinical Study?	☐ Yes ☐ No<br>If yes, please provide the following information: |__| Sponsor-Initiated Clinical Study |__| Investigator-Initiated Clinical Study
Has the clinical case been presented to congresses/poster sessions/etc..	☐ Yes ☐ No<br>If yes, please provide information:<br>Congress year ___________________________<br>Congress Event Name _____________________
Additional Notes	— — — — — — — —

**Adverse Event Status**			
☐	Closed/Resolved	Start date	|__|__| |__|__| |__|__|__|__| dd mm yyyy
		Stop date	|__|__| |__|__| |__|__|__|__| dd mm yyyy
☐	Closed (Stop date unknown)		
☐	Still ongoing/in progress		

Further details and comments:

____

____

Date of completion: |__|__| |__|__| |__|__|__|__| Signature: ____________________________
dd mm yyyy
